# LncRNA XIST sponges miR-199a-3p to modulate the Sp1/LRRK2 signal pathway to accelerate Parkinson’s disease progression

**DOI:** 10.18632/aging.202378

**Published:** 2021-01-20

**Authors:** Qian Zhou, Ming-Ming Zhang, Min Liu, Zhi-Gang Tan, Qi-Lin Qin, Yu-Gang Jiang

**Affiliations:** 1 Department of Neurosurgery, Second Xiangya Hospital of Central South University, Changsha 410011, Hunan Province, P.R. China

**Keywords:** Parkinson's disease, lncRNA XIST, miR-199a-3p, Sp1, LRRK2

## Abstract

*In vitro* and *in vivo* models of Parkinson’s disease were established to investigate the effects of the lncRNA XIST/miR-199a-3p/Sp1/LRRK2 axis. The binding between XIST and miR-199a-3p as well as miR-199a-3p and Sp1 were examined by luciferase reporter assay and confirmed by RNA immunoprecipitation analysis. Following the Parkinson’s disease animal behavioural assessment by suspension and swim tests, the brain tissue injuries were evaluated by hematoxylin and eosin, TdT-mediated dUTP-biotin nick end labelling, and tyrosine hydroxylase stainings. The results indicated that miR-199a-3p expression was downregulated, whereas that of XIST, Sp1 and LRRK2 were upregulated in Parkinson’s disease. Moreover, miR-199a-3p overexpression or XIST knockdown inhibited the cell apoptosis induced by MPP^+^ treatment and promoted cell proliferation. The neurodegenerative defects were significantly recovered by treating the cells with shXIST or shSp1, whereas miR-199a-3p inhibition or Sp1 and LRRK2 overexpression abrogated these beneficial effects. Furthermore, the results of our *in vivo* experiments confirmed the neuroprotective effects of shXIST and miR-199a-3p against MPTP-induced brain injuries, and the Parkinson’s disease behavioural symptoms were effectively alleviated upon shXIST or miR-199a-3p treatment. In summary, the results of the present study showed that lncRNA XIST sponges miR-199a-3p to modulate Sp1 expression and further accelerates Parkinson’s disease progression by targeting LRRK2.

## INTRODUCTION

Parkinson’s disease (PD) is a neurodegenerative disorder that exhibits evolving layers of complexity over the long term, and its prevalence ranges from 1-2 per 1000 individuals, affecting seven to ten million people worldwide [1]. PD is clinically characterized by the elevated loss of substantia nigra pars compacta (SNpc) dopaminergic neurons and the disruption of motor functions. As the disease worsens, the patients suffer from bradykinesia, postural instability, resting tremor and muscular rigidity [2]. Although it is generally believed that genetic and environmental risk factors play important roles in PD development by damaging various fundamental cellular processes [3], the exact cause of PD in most remains unknown. Thus, a better understanding of the underlying mechanism would potentially contribute to the development of more effective therapeutic methods for PD.

Leucine-rich repeat kinase 2 (LRRK2) is a 286 kDa multifunctional protein kinase that consists of armadillo repeats [4], ankyrin repeats (ANK), leucine-rich repeats (LRR), a Rho/Ras-like GTPase, and a WD40 domain [5]. Over 100 mutations in the LRRK2 gene have been demonstrated to be associated with PD development [6] by preventing mitochondrial fission [7], attenuating microglial motility [8], and increasing neural death [9–12]. Furthermore, accumulating evidence has shown that LRRK2 is overexpressed in PD, and LRRK2 mutation is the most common cause of PD identified to date [12–14]. Transcription factor synphilin-1 (Sp1) binds to various promoters at their GC-rich motifs and is involved in many cellular processes as well as in PD [15, 16]. Studies have shown that LRRK2 gene promoter activity and gene expression are promoted by the Sp1 signalling pathway [17]. In addition, it mutant LRRK2-induced neurodegeneration has been reported to be inhibited by Sp1 in a PD model [18]. In the present study, we observed that Sp1 was upregulated in PD and promoted LRRK2 expression, in accordance with the findings of previous studies. However, the upstream regulatory mechanism of LRRK2 and Sp1 in PD progression remains to be further elucidated.

MicroRNAs (miRNAs) are small (approximately 22 nucleotides in length) non-coding RNAs that function in RNA silencing and gene expression regulation at the posttranscriptional level by binding target messenger RNAs (mRNAs) at their 3’-untranslated region (3’-UTR). MiRNAs including miR-128, miR-7, miR-27a/27b and miR-200a were reported to be aberrantly expressed during PD progression and play crucial roles in PD pathogenesis by repressing [19–22] or promoting dopamine neuron apoptosis [4, 23, 24]. A number of these miRNAs have been demonstrated to directly target Sp1 in different diseases [25–27]. For example, a previous study showed that Sp1 is a downstream target of miR-199a-3p in testicular germ cell tumours [28]. MiR-199a-3p has been reported as a tumour suppressor in various human cancers, including breast cancer [29], osteosarcoma [30] and colorectal cancer [31]. In the present study, our bioinformatics analysis results predicted miR-199a-3p as a Sp1 binding partner, and it was shown to significantly inhibit Sp1 expression. However, an understanding of the molecular expression and function of miR-199a-3p in PD pathogenesis has remained elusive, especially with respect to how it regulates LRRK2 by interacting with Sp1.

Long non-coding RNAs (lncRNAs) are transcribed RNAs that are longer than 200 nucleotides and do not encode proteins. Recently, there has been increased interest in lncRNAs in brain development research and neurodegenerative diseases [32]. Abnormal expression of lncRNAs including NEAT1 [33], HOTAIR [34], SNHG1 [35], MALAT1 [36] and p21 [37] was detected in PD cellular or animal models. The lncRNA XIST (X-inactive specific transcript) is derived from the XIST gene and was reported to be a key regulator in cell proliferation and differentiation by interacting with its binding partners [38]. However, the expression level and molecular role of lncRNA XIST in PD have yet to be elucidated. Using bioinformatics analysis, we speculated that the lncRNA XIST is a potential binding partner of miR-199a-3p and subsequently showed that lncRNA XIST directly inhibits miR-199a-3p expression. However, the regulatory association between lncRNA XIST and miR-199a-3p has not been reported to date.

In the present study, we provide evidence that lncRNA XIST sponges miR-199a-3p and then enhances the expression of Sp1 and LRRK2, thereby accelerating PD progression. The results of our study expands our knowledge of PD aetiologies and highlights lncRNA XIST and miR-199a-3p as promising therapeutic targets for PD prevention.

## RESULTS

### LncRNA XIST, miR-199a-3p, Sp1 and LRRK2 expression in a PD *in vitro* model

**Figure 1:**
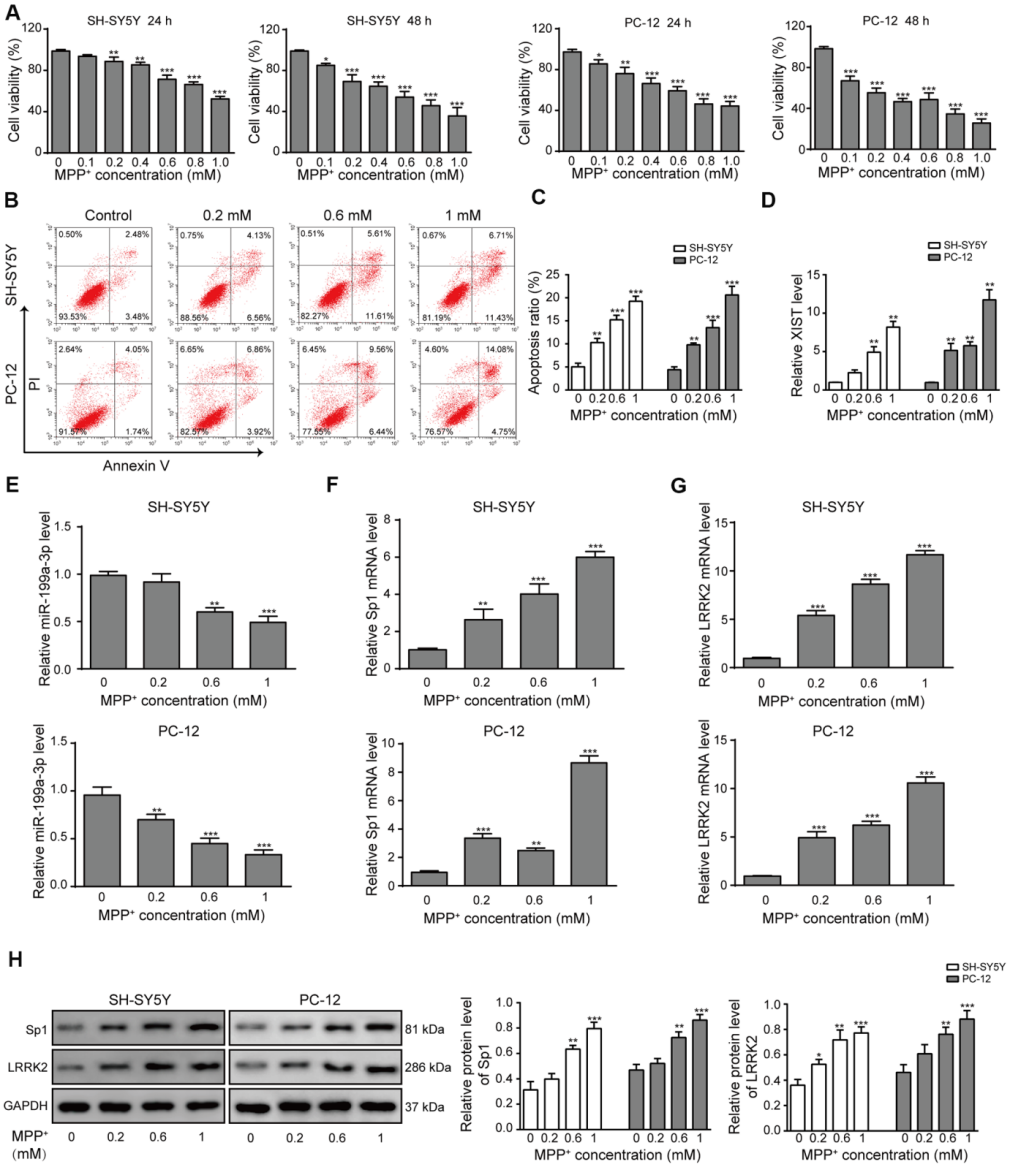
**LncRNA XIST, miR-199a-3p, Sp1 and LRRK2 expression in an *in vitro* model of PD.** (**A**) MPP^+^ was added to the cells to final concentrations of 0, 0.1, 0.2, 0.4, 0.6, 0.8 or 1 mM. Cell viability was determined using the CCK-8 assay after 24 and 48 h. (**B**) Flow cytometry analysis was performed to measure the apoptosis of SH-SY5Y and PC-12 cells, which were treated with 0, 0.2, 0.6, or 1 mM of MPP^+^. (**C**) Comparison of apoptotic cells in different groups. (**D**–**G**) Relative expression of (**D**) XIST, (**E**) miR-199a-3p, (**F**) Sp1 mRNA and (**G**) LRRK2 mRNA in the above groups of cells were determined by qPCR analysis. (**H**) Western blot results showed that the protein levels of Sp1, LRRK2 were elevated when the cells were treated with MPP^+^. GAPDH was used as a loading control. The data are representative of three experiments. *p <0.05, **p <0.01 and ***p <0.001.

We postulated that the steady-state expression of XIST, miR-199a-3p, Sp1 and LRRK2 are altered during PD progression and used MPP+ to generate *in vitro* PD model in SH-SY5Y and PC-12 cells. Cell proliferation decreased in an MPP+ concentration-dependent manner in both cell lines (Figure 1A). Flow cytometry scatter plots also showed a trend of decreased cell survival and increased cell apoptosis with increasing MPP+ concentration (Figure 1B, 1C). When the MPP+ concentration was increased, the level of miR-199a-3p expression decreased (Figure 1E), and the levels of XIST (Figure 1D), Sp1 mRNA (Figure 1F) and LRRK2 mRNA (Figure 1G) expression increased. The western blot results also revealed increased protein expression of Sp1 and LRRK2 (Figure 1H). These results indicated that the levels of XIST, miR-199a-3p, Sp1 and LRRK2 expression may be associated with PD pathogenesis and its further development.

### MiR-199a-3p overexpression prevents MPP^+^-induced cellular toxicity

**Figure 2:**
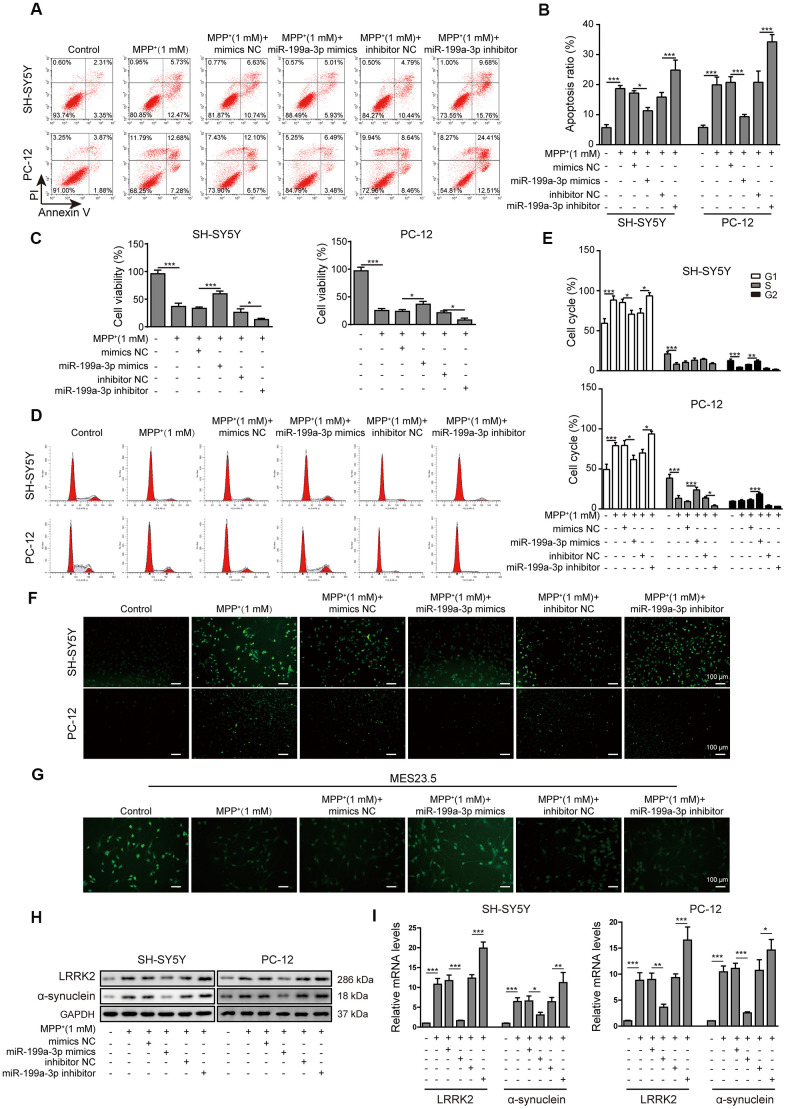
**MiR-199a-3p overexpression prevents MPP^+^-induced cellular toxicity.** (**A**) The flow cytometry assay was performed to evaluate the apoptosis of SH-SY5Y and PC-12 cells that were treated with 1 mM MPP^+^ and transfected with the miR-199a-3p mimics, miR-199a-3p inhibitor, mimics NC or inhibitor NC. (**B**) Apoptotic cells in the different groups were compared. (**C**) The CCK-8 assay was performed to evaluate the viability of SH-SY5Y and PC-12 cells. (**D**) The cell cycle phases were determined by propidium iodide staining and flow cytometry. (**E**) Comparison of the cell cycle analysis results for the different groups. (**F**) TUNEL staining was conducted to assess SH-SY5Y and PC-12 cell apoptosis. (**G**) Immunofluorescence staining was performed to monitor the distribution of TUBB3 in MES23.5 cells. (**H**) Western blot and (**I**) qPCR results showed that miR-199a-3p overexpression reduced LRRK2 and α-synuclein expression at both the protein and mRNA levels, which were promoted by MPP^+^ treatment. The data are representative of three experiments. *p <0.05, **p <0.01 and ***p <0.001.

To examine the function of miR-199a-3p during PD progression, we transfected SH-SY5Y and PC-12 cells with miR-199a-3p mimics or a miR-199a-3p inhibitor, the efficiency of which was assessed by qPCR (Supplementary Figure 1A). The relative expression of miR-199a-3p was measured after the cells were treated with MPP+ and miR-199a-3p mimics or inhibitor transfection. MiR-199a-3p mimics caused the upregulation while miR-199a-3p inhibitor further caused the downregulation of miR-199a-3p induced by MPP+ (Supplementary Figure 1B). Cell apoptosis was significantly decreased in the miR-199a-3p overexpression group and increased in miR-199a-3p inhibition group compared to that observed in the control groups (Figure 2A, 2B). The TUNEL staining (Figure 2F) results further confirmed the protective function of miR-199a-3p on cell apoptosis in both cell lines treated with MPP+. Cell viability was also significantly increased in the miR-199a-3p overexpression group and decreased in miR-199a-3p inhibition group compared to that observed in the control groups (Figure 2C). A similar protective effect of miR-199a-3p was observed in our cell cycle analysis experiment. These results indicated that MPP+ treatment induced cell cycle arrest at G1 phase, miR-199a-3p overexpression reversed this phenomenon while miR-199a-3p inhibition reinforced it (Figure 2D, 2E). To investigate the function of miR-199a-3p in a neuronal context, we stained MES23.5 cells using a fluorescent-labelled antibody against TUBB3. MPP+-treated MES23.5 cells displayed decreased TUBB3 expression, which was reversed by miR-199a-3p overexpression and further strengthened by miR-199a-3p inhibition (Figure 2G). As the results of previous studies indicated that LRRK2 is significantly associated with the onset of PD [39], we tested whether miR-199a-3p impacted LRRK2 and another PD marker α-synuclein expression in the present study. The results of western blot (Figure 2H) and qPCR (Figure 2I) analyses showed that miR-199a-3p overexpression inhibited LRRK2 and α-synuclein expression at both the protein and mRNA levels, which was promoted by MPP+ treatment. This effect was notably reversed in the cells transfected with the miR-199a-3p inhibitor. Taken together, these results indicated that miR-199a-3p overexpression reduced LRRK2 and α-synuclein expression and protected cells from the cellular toxicity induced by MPP+.

### XIST directly targets miR-199a-3p

**Figure 3:**
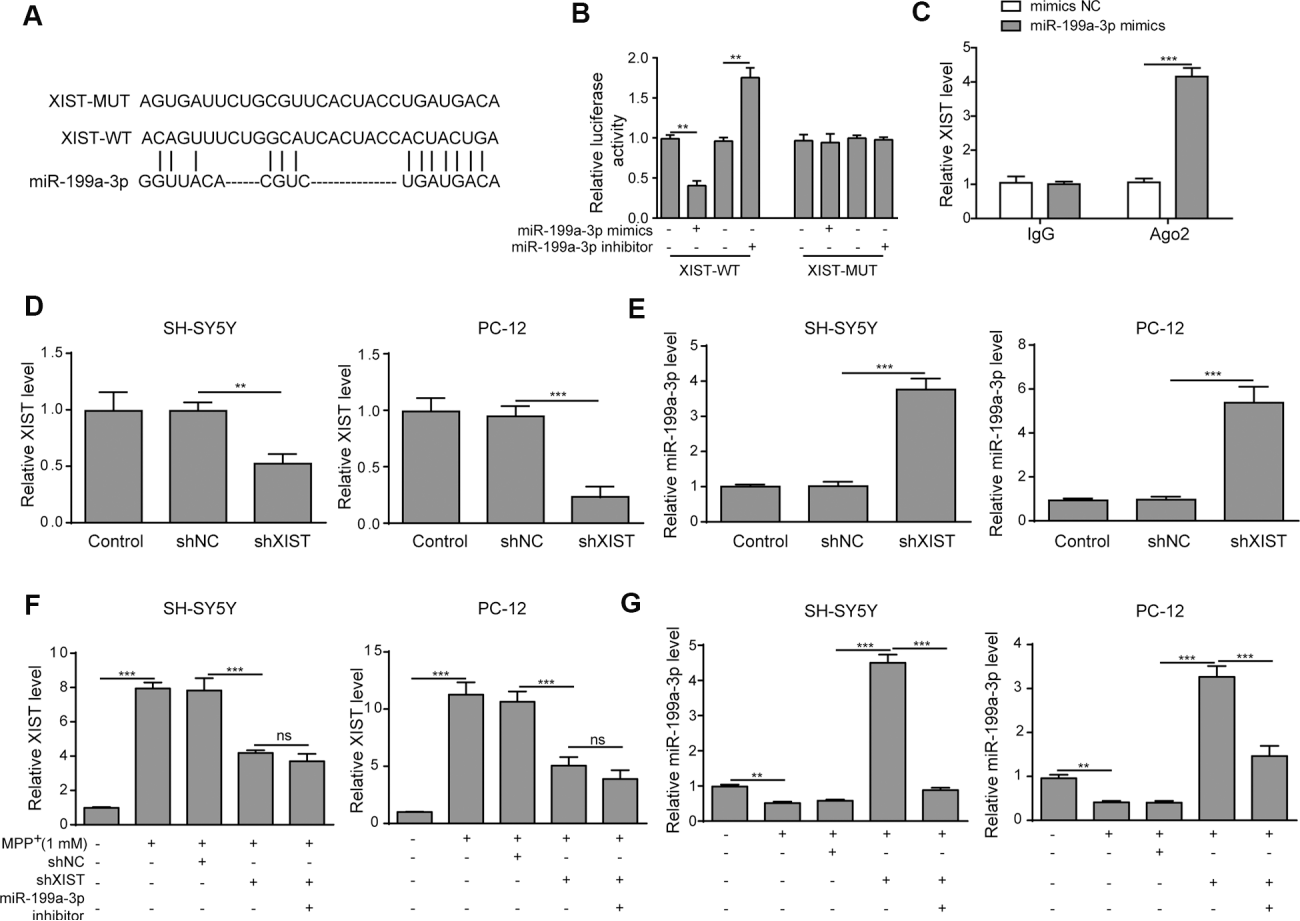
**XIST directly targets miR-199a-3p.** (**A**) The putative binding sites of miR-199a-3p in XIST were predicted using DIANA tools. (**B**) HEK-293T cells were co-transfected with the miR-199a-3p mimics or inhibitor and XIST-WT or XIST-MUT luciferase reporter plasmids. Luciferase activities were measured 48 h after transfection. (**C**) The binding between miR-199a-3p and XIST was further confirmed by RIP analysis. (**D**) SH-SY5Y and PC-12 cells were transfected with shXIST. Transfection efficiency was assessed by qPCR analysis. (**E**) The effect of shXIST on miR-199a-3p expression was evaluated by qPCR. (**F**) XIST expression was inhibited by transfecting the SH-SY5Y and PC-12 cells with shXIST. (**G**) qPCR results showed that shXIST transfection improved miR-199a-3p expression, which was repressed by co-transfection with the miR-199a-3p inhibitor. The data are representative of three experiments. **p <0.01, ***p <0.001 and ns, not significant.

To evaluate the relationship between XIST and miR-199a-3p, we first identified several potential target genes using DIANA tools. The predicted binding sites between XIST and miR-199a-3p were shown in Figure 3A. To confirm the regulatory effect of XIST in miR-199a-3p expression, the putative miR-199a-3p binding site in the wild-type XIST sequence (XIST-WT) or its corresponding mutant (XIST-MUT) was inserted a firefly luciferase reporter gene. The results showed that the relative luciferase activity of XIST-WT construct was greatly reduced by miR-199a-3p mimics transfection and increased by miR-199a-3p inhibition. In contrast, the relative luciferase activity of the XIST-MUT construct displayed no changes upon transfection of the miR-199a-3p mimics or inhibitor (Figure 3B). Consistent with these findings, our RIP results showed a great increase in co-precipitated XIST levels after cells were treated with miR-199a-3p mimics compared to that observed in the negative control (Figure 3C). These results confirmed the binding between XIST and miR-199a-3p. Next, we transfected SH-SY5Y and PC-12 cells with shXIST, and the transfection efficiency was assessed and shown in Figure 3D. The effect of shXIST on miR-199a-3p expression was evaluated by qPCR analysis and results indicated that shXIST treatment caused the upregulation of miR-199a-3p (Figure 3E). We also observed that XIST expression was efficiently inhibited by shXIST in the MPP^**+**^-treated cells (Figure 3F). In addition, the qPCR results showed that miR-199a-3p expression was promoted by shXIST and decreased by co-transfection with the miR-199a-3p inhibitor (Figure 3G). Taken together, our results demonstrated that miR-199a-3p is a direct binding target of XIST.

### XIST knockdown reduces MPP^+^-induced cellular damages by directly targeting miR-199a-3p

**Figure 4:**
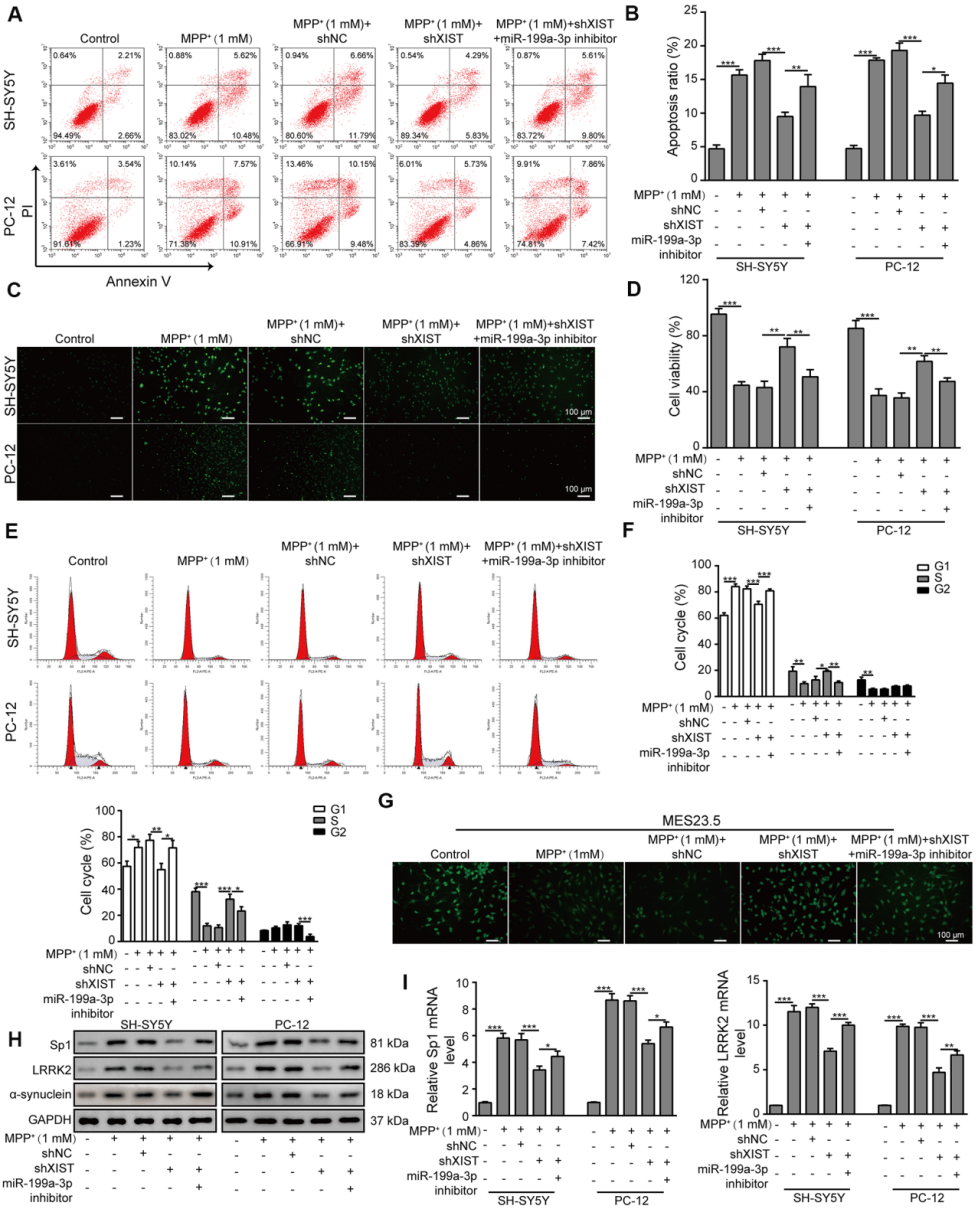
**XIST knockdown reduces MPP^+^-induced cellular damage by directly targeting miR-199a-3p.** (**A**) Flow cytometry analysis of apoptosis was performed upon the silencing of XIST and/or miR-199a-3p. (**B**) Apoptotic cells in different groups were compared. (**C**) TUNEL staining was performed to evaluate cell apoptosis. (**D**) The viability of SH-SY5Y and PC-12 cells transfected or treated with the indicated molecules was evaluated by the CCK-8 assay. (**E**) The cell cycle phases were determined by propidium iodide staining and flow cytometry after the cells were treated with shXIST and/or miR-199a-3p inhibitor. (**F**) Comparison of the cell cycle results for the different groups. (**G**) Immunofluorescence staining of TUBB3 in MES23.5 cells is shown. (**H**) Western blot and (**I**) qPCR results showed that the inhibitory effects of shXIST on Sp1, LRRK2 and α-synuclein expression were counteracted by miR-199a-3p knockdown. The data are representative of three experiments. *p <0.05, **p <0.01 and ***p <0.001.

Next, we evaluated the impact of shXIST and the miR-199a-3p inhibitor on cell apoptosis in the SH-SY5Y and PC-12 cell lines. Interestingly, both cell lines treated with shXIST displayed significantly reduced apoptosis rates compared to those observed in cells treated with MPP^+^ and shNC. In contrast, co-transfection with the miR-199a-3p inhibitor and shXIST increased the cellular apoptosis of both cell lines (Figure 4A, 4B). To substantiate these observations, we further assessed the cell apoptosis rate by TUNEL staining (Figure 4C) and cell proliferation by CCK-8 assay (Figure 4D). The inhibitory effects of shXIST on MPP^**+**^-induced apoptosis in both SH-SY5Y and PC12 cells were almost completely abrogated by the inhibition of miR-199a-3p. Consistent with these findings, we observed similar results in our cell cycle analysis experiments, which showed that shXIST inhibited cell cycle arrest at G1 phase, while miR-199a-3p inhibition reversed these effects (Figure 4E, 4F). We then tested whether these effects were relevant in a neuronal context by labelling MES23.5 cells with a TUBB3 antibody (Figure 4G). Notably, shXIST restored TUBB3 expression in MPP**^+^-**treated cells, and this effect was counteracted by transfection with the miR-199a-3p inhibitor. Furthermore, the Sp1, LRRK2 and α-synuclein protein levels were downregulated by shXIST but upregulated by co-transfection with shXIST and the miR-199a-3p inhibitor (Figure 4H). The mRNA levels of Sp1 and LRRK2 showed the similar changes (Figure 4I). These results suggested that shXIST protected cells against MPP^**+**^-induced neuronal damage by directly targeting miR-199a-3p, and this protective effect was reversed by the miR-199a-3p inhibitor.

### Sp1 is a direct target of miR-199a-3p

**Figure 5:**
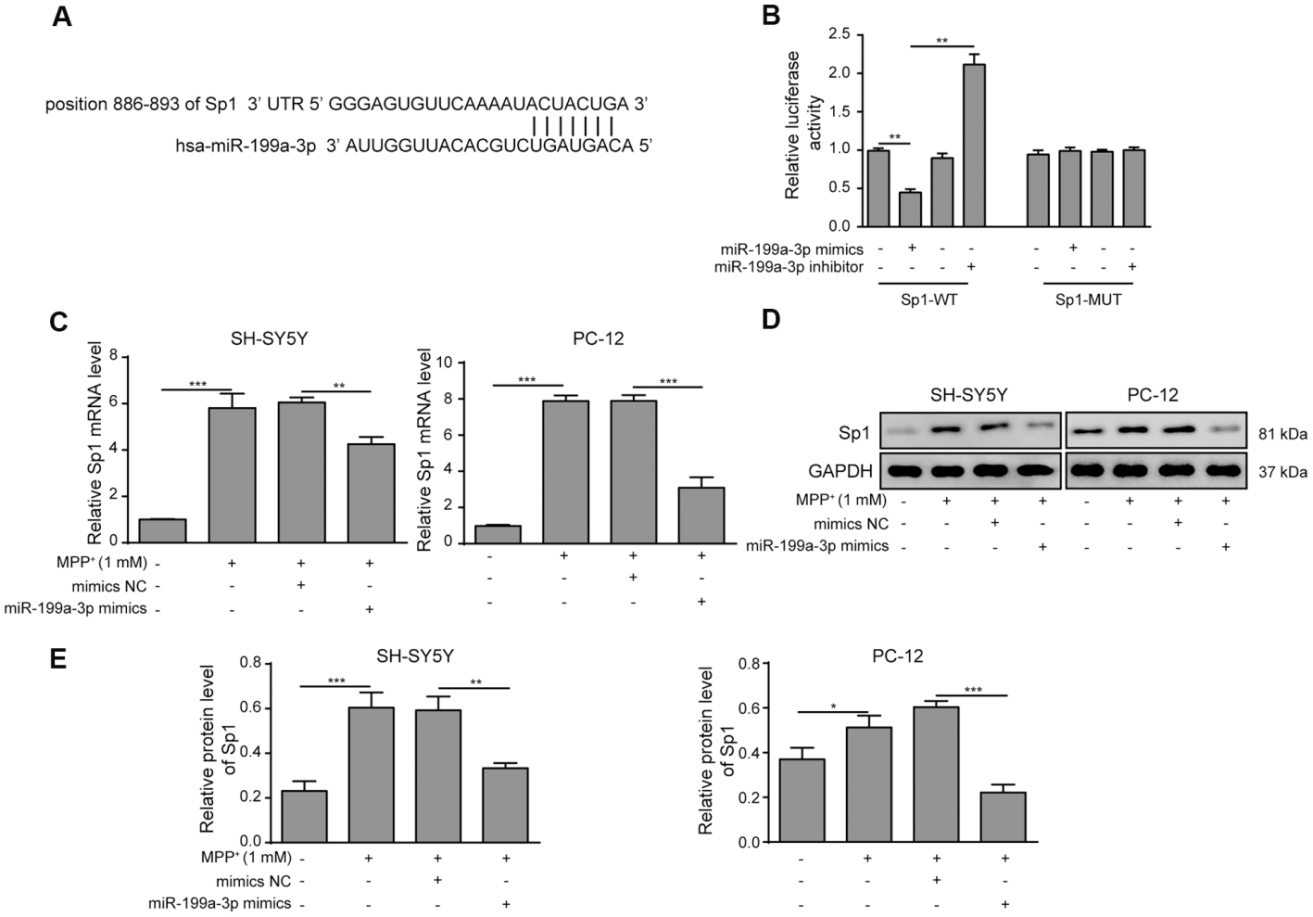
**Sp1 is a direct target of miR-199a-3p.** (**A**) The potential binding sites of miR-199a-3p in Sp1 mRNA were predicted by TargetScan. (**B**) HEK-293T cells were co-transfected with the miR-199a-3p mimics or inhibitor and Sp1-WT or Sp1-MUT luciferase reporter plasmids. The cells were harvested 48 h after transfection for luciferase activity determination. (**C**) qPCR results showed that the upregulating effect of MPP^+^ on Sp1 mRNA expression was inhibited by miR-199a-3p overexpression. (**D**, **E**) Western blot analysis was performed to measure the protein expression of Sp1 upon the miR-199a-3p transfection. The data are representative of three experiments. *p <0.05, **p <0.01 and ***p <0.001.

To further evaluate the association between miR-199a-3p and Sp1, we next showed that Sp1 is a potential target gene of miR-199a-3p using TargetScan tools. The putative binding sites between miR-199a-3p and Sp1 were shown in Figure 5A. To evaluate this potential binding, we performed luciferase reporter assays by inserting the predicted miR-199a-3p targeting site of Sp1 (Sp1-WT) or its corresponding mutant (Sp1-MUT) to a firefly luciferase reporter gene. As shown in Figure 5B, Sp1-WT luciferase activity was markedly reduced by miR-199a-3p co-expression and promoted by the miR-199a-3p inhibitor. In contrast, no changes were observed in the Sp1-MUT groups. These results confirmed that Sp1 is a target of miR-199a-3p and prompted us to assess the impact of miR-199a-3p on Sp1 expression in a PD cellular model. In addition, through qPCR and western blot analyses, we observed that miR-199a-3p overexpression largely reduced Sp1 expression at both the mRNA (Figure 5C) and protein (Figure 5D, 5E) levels that had been induced by MPP^**+**^ treatment.

### Neuroprotective effects of shSp1 are suppressed by miR-199a-3p inhibition

**Figure 6:**
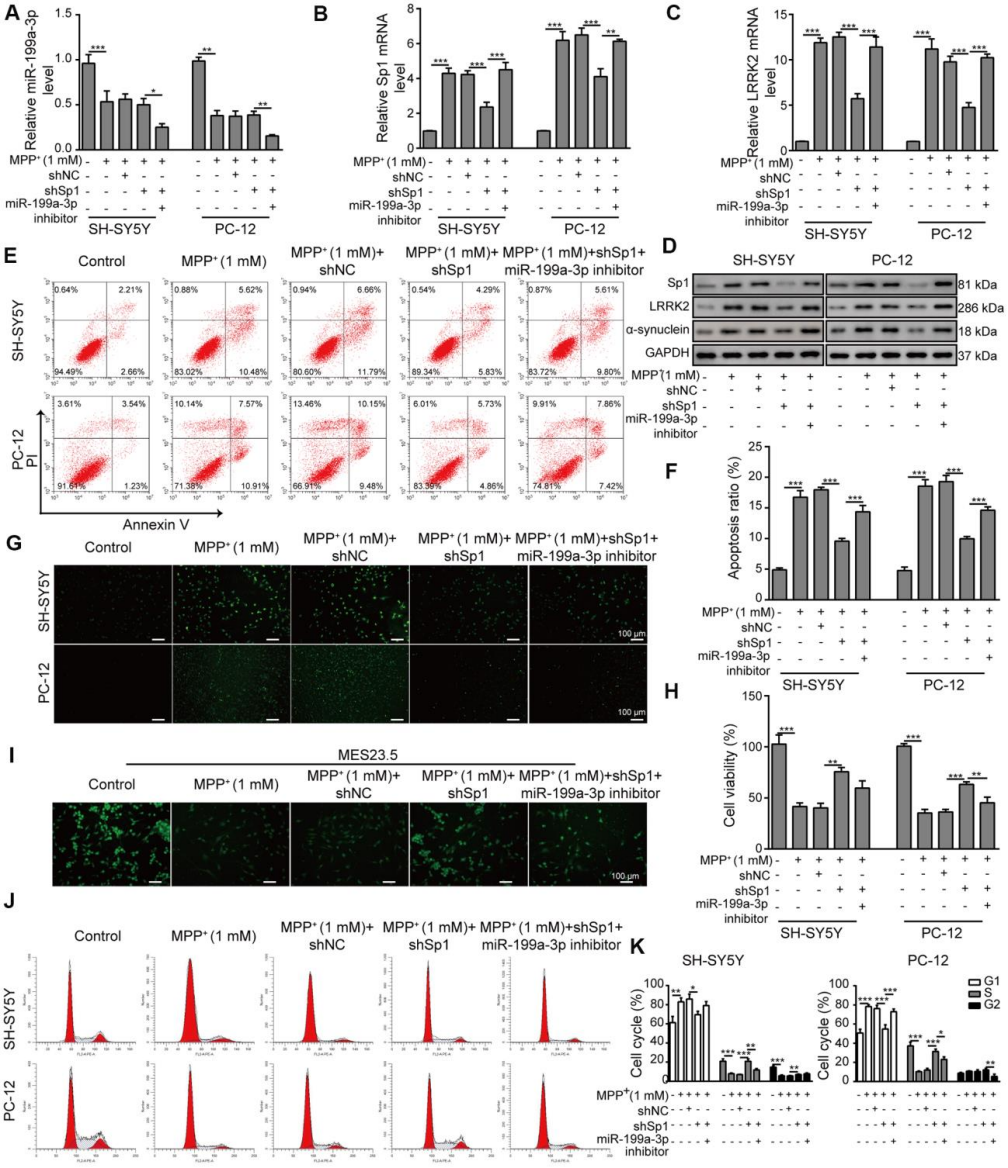
**Neuroprotective effects of shSp1 are suppressed by miR-199a-3p inhibition.** SH-SY5Y and PC-12 cells were treated with 1 mM MPP^+^ after transfection with shSp1 or the miR-199a-3p inhibitor. qPCR was performed to assess the expression of (**A**) miR-199a-3p, (**B**) Sp1 mRNA, and (**C**) LRRK2 mRNA. (**D**) Western blot analysis was conducted to measure the protein expression of Sp1, LRRK2 and α-synuclein under the indicated conditions. (**E**) Flow cytometry analysis of apoptosis was performed after the cells were transfected with shSp1 and/or the miR-199a-3p inhibitor. (**F**) Comparison of apoptotic cells in the indicated groups. (**G**) TUNEL staining was performed to assess cell apoptosis. (**H**) Cell viability was determined by the CCK-8 assay after the cells were transfected with shSp1 alone or co-transfected with the miR-199a-3p inhibitor. (**I**) MES23.5 cells were transfected with shNC, shSp1 or the miR-199a-3p inhibitor and treated with 1 mM MPP^+^. The localization of TUBB3 in MES23.5 cells is shown in the representative images. (**J**) Cell cycle phases were determined by propidium iodide staining and flow cytometry. (**K**) Comparison of the cell cycle for the different groups. The data are representative of three experiments. *p <0.05, **p <0.01 and ***p <0.001.

We next transfected SH-SY5Y and PC-12 cells with the miR-199a-3p inhibitor or shSp1 and investigated the regulatory effect of miR-199a-3p on Sp1. The transfection efficiency of shSp1 was confirmed by qPCR (Supplementary Figure 2A). The results showed that miR-199a-3p expression was not altered by shSp1 transfection in MPP^**+**^-treated cells (Figure 6A), whereas the suppressive effect of shSp1 on Sp1 expression was markedly reversed by miR-199a-3p inhibition (Figure 6B). Intriguingly, LRRK2 expression was markedly lower in the cells transfected with shSp1 compared to that observed in cells treated with MPP^**+**^ or shNC alone (Figure 6C), while co-transfection of miR-199a-3p inhibitor with shSp1 restored LRRK2 and α-synuclein expression at protein level (Figure 6D). The results of a previous study showed that SH-SY5Y cells could be rescued from MPP^**+**^-induced cellular apoptosis by Sp1 inhibition [15]. Interestingly, in the present study, we observed that the protection of shSp1 against MPP^**+**^-induced apoptosis was completely abrogated by miR-199a-3p inhibition (Figure 6E–6H). Additionally, MPP^**+**^ treatment notably reduced TUBB3 expression compared to that observed in the healthy MES23.5 cells. In contrast, Sp1 inhibition largely restored TUBB3 expression in MES23.5 cells (Figure 6I). However, cells that were co-transfected with shSp1 and the miR-199a-3p inhibitor exhibited lower TUBB3 expression than those transfected with shSp1 alone (Figure 6I). Similar interactive effects were observed in our cell cycle analysis experiments (Figure 6J, 6K). Taken together, these results demonstrated that miR-199a-3p directly targets Sp1 and downregulated its expression in a PD model, thereby preventing Sp1 from exacerbating cellular defects.

### Sp1 overexpression inhibits the neural protective function of shXIST and aggravates MPP^+^-induced neurodegeneration

**Figure 7:**
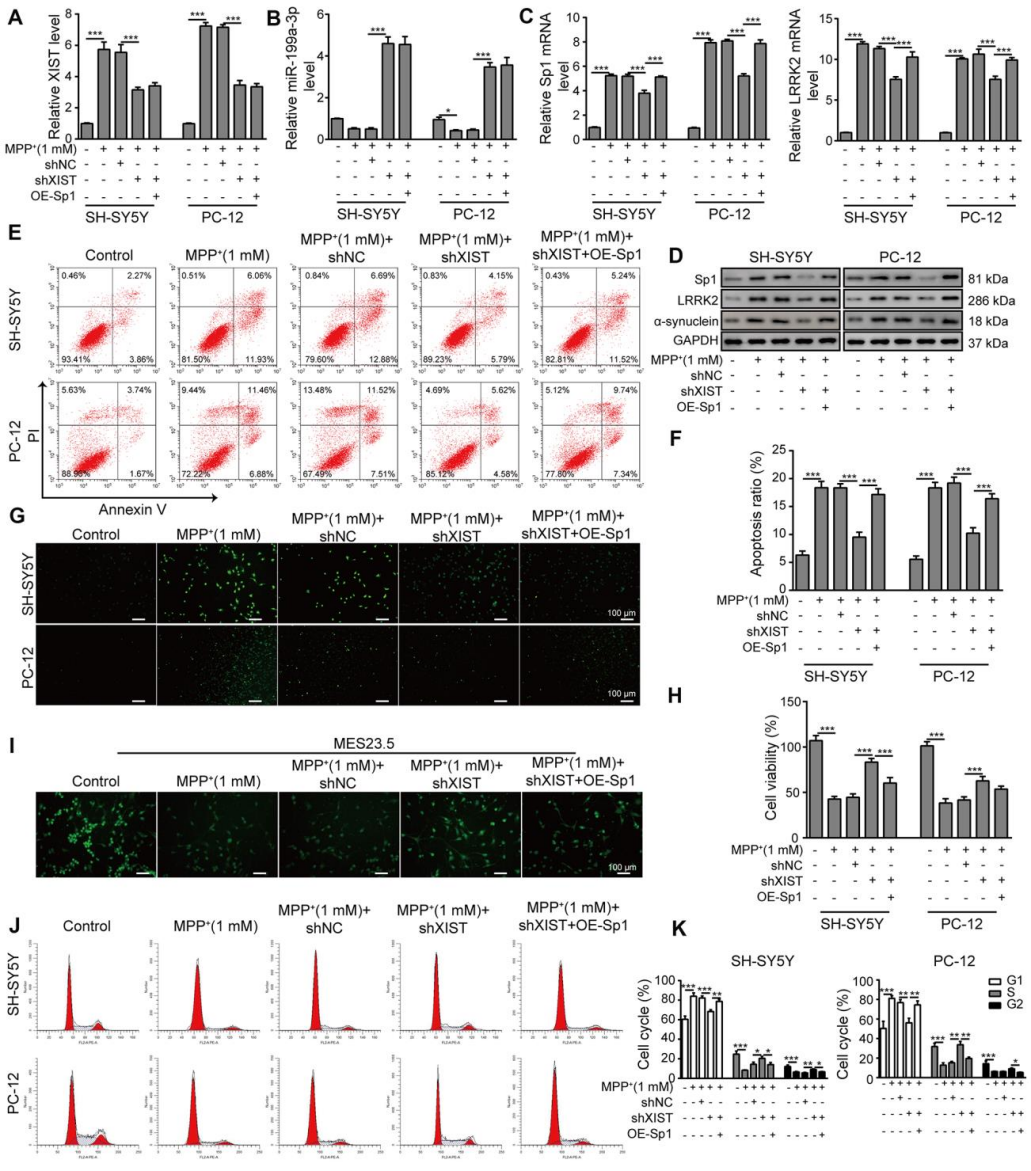
**Sp1 overexpression inhibits the neural protective function of shXIST and aggravates MPP^+^-induced neurodegeneration.** SH-SY5Y and PC-12 cells were transfected with shNC, shXIST or the Sp1 overexpression vector and treated with 1 mM MPP^+^. The expression of (**A**) XIST, (**B**) miR-199a-3p, (**C**) Sp1 and LRRK2 mRNA was determined by qPCR analysis. (**D**) Western blot analysis was performed to analyse the protein expression of Sp1, LRRK2 and α-synuclein under the specific conditions. (**E**) Flow cytometry analysis of apoptosis was performed after the cells were transfected with shXIST and/or Sp1. (**F**) Comparison of apoptotic cells in the indicated groups. (**G**) TUNEL staining was performed to measure the apoptosis rate of the SH-SY5Y and PC-12 cells after they were transfected with shXIST alone or co-transfected with Sp1. (**H**) The CCK-8 assay was performed to assess cell viability. (**I**) MES23.5 cells were transfected with shNC, shXIST or Sp1 and treated with 1 mM MPP^+^. The localization of TUBB3 in MES23.5 cells is shown in the representative images. (**J**) Cell cycle phases were determined by propidium iodide staining and flow cytometry after the cells were transfected with shXIST alone or co-transfected with Sp1. (**K**) The cell cycle results for the different groups were compared. The data are representative of three experiments. *p <0.05, **p <0.01 and ***p <0.001.

To investigate the relationship between XIST and Sp1, we overexpressed Sp1 in SH-SY5Y and PC-12 cells that were co-transfected with shXIST. The transfection efficiency of OE-Sp1 (overexpression of Sp1) was evaluated by qPCR (Supplementary Figure 3A). In the MPP^+^-treated cells, the expression of XIST (Figure 7A) and miR-199a-3p (Figure 7B) was significantly inhibited or promoted by shXIST but not by Sp1 overexpression. Notably, the mRNA expressions of both Sp1 and LRRK2 in the shXIST group were largely reduced compared to that observed in the MPP^+^ group and were almost completely restored upon Sp1 overexpression (Figure 7C). Additionally, we measured the protein expression of Sp1, LRRK2 and α-synuclein by western blot analysis and obtained consistent results (Figure 7D). To further study whether shXIST and Sp1 overexpression can alter MPP**^+^-**induced cell apoptosis, we next performed flow cytometry analysis (Figure 7E, 7F). Compared to the cells treated with MPP^**+**^ alone, cells transfected with shXIST displayed a significantly decreased apoptosis rate, whereas this protective effect was suppressed by Sp1 overexpression. To further substantiate this observation, we next performed TUNEL staining (Figure 7G) as well as CCK-8 assays (Figure 7H) and observed consistent results. To test whether these findings are relevant in a neuronal context, we labelled MES23.5 cells using a TUBB3 antibody. Compared to the MPP^+^ and MPP^+^+shNC groups, the treatment of shXIST greatly enhanced TUBB3 expression, and this effect was almost completely abolished by Sp1 overexpression (Figure 7I). Finally, our cell cycle analysis, as assessed by propidium iodide staining and flow cytometry, further confirmed the interactive effects of shXIST and Sp1 overexpression on MPP**^+^-**induced cellular damage (Figure 7J, 7K). Taken together, these results demonstrated that Sp1 is a crucial downstream regulatory molecule of XIST.

### LRRK2 overexpression counteracts the neural protective effect of shSp1

**Figure 8:**
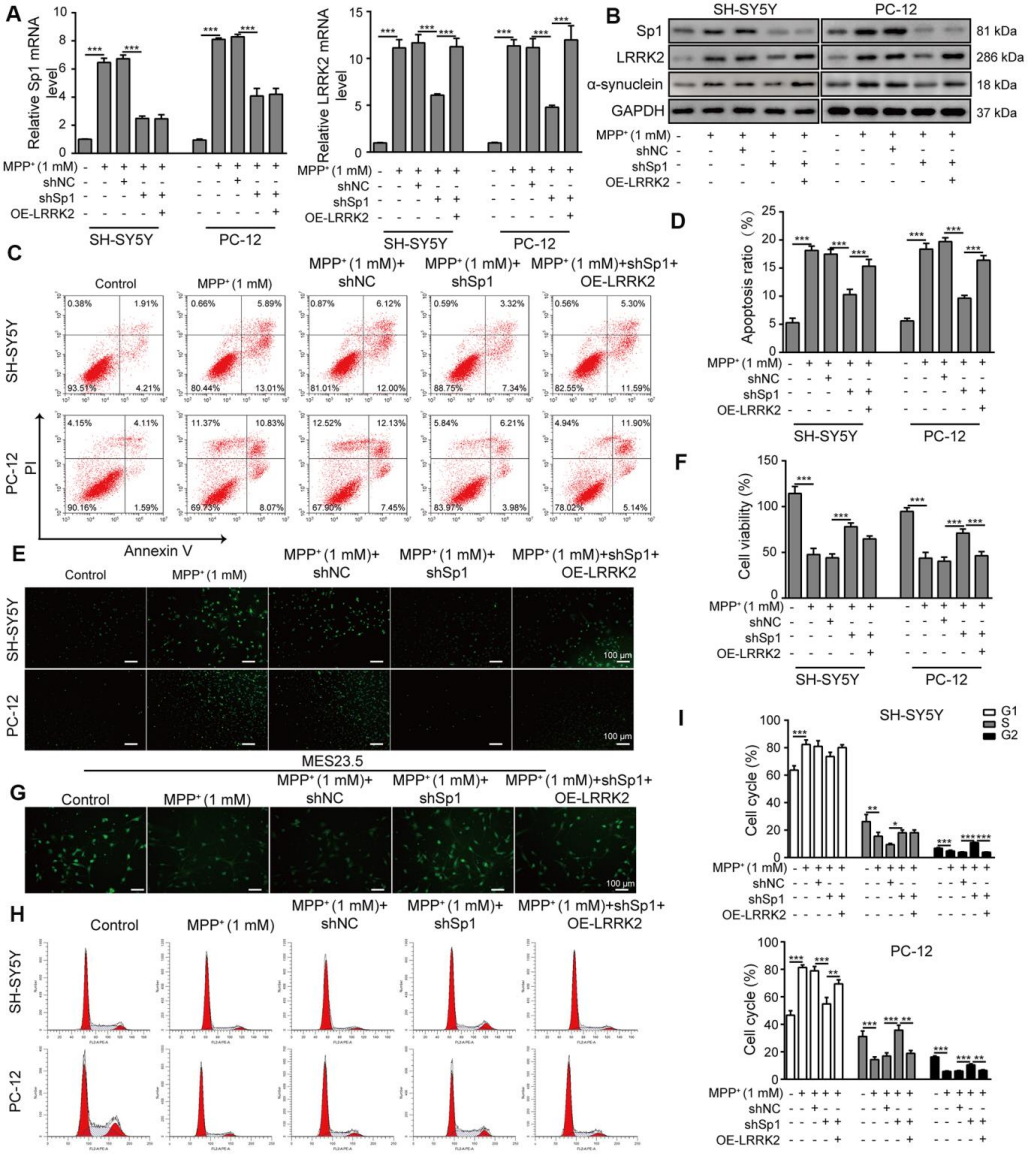
**LRRK2 overexpression counteracts the neural protective effect of shSp1.** SH-SY5Y and PC-12 cells were treated with 1 mM MPP^+^ and transfected with shNC, shSp1 or the LRRK2 overexpression vector. (**A**) The expressions of Sp1 and LRRK2 at the mRNA level were determined by qPCR analysis. (**B**) The expressions of Sp1, LRRK2 and α-synuclein at protein level were determined by western blot analysis. (**C**) Flow cytometry analysis of apoptosis was performed. (**D**) Apoptotic cells in different groups were compared. (**E**) TUNEL staining was performed to assess SH-SY5Y and PC-12 cell apoptosis under the indicated conditions. (**F**) Cell proliferation was determined by the CCK-8 assay. (**G**) MES23.5 cells were transfected with shNC, shSp1 or LRRK2 overexpression vector and treated with 1 mM MPP^+^. The localization of TUBB3 in MES23.5 cells is shown in the representative images. (**H**) Cell cycle phases were determined by propidium iodide staining and flow cytometry. (**I**) Comparison of cell cycle of SH-SY5Y and PC-12 cells in the different groups. The data are representative of three experiments. *p <0.05, **p <0.01 and ***p <0.001.

We transfected SH-SY5Y and PC-12 cells with OE-LRRK2 or OE-NC and assessed the transfection efficiency by qPCR (Supplementary Figure 4A). To verify the interaction between LRRK2 and Sp1, we overexpressed LRRK2 in SH-SY5Y and PC-12 cells that were co-transfected with shSp1. As shown in Figure 8A, 8B, shSp1 treatment greatly inhibited not only the expression of Sp1 but also LRRK2 and α-synuclein expression levels. In addition, the inhibitory effect of shSp1 on LRRK2 and α-synuclein was effectively abrogated by LRRK2 overexpression. However, the inhibition of Sp1 expression by shSp1 treatment was not altered upon LRRK2 overexpression. Moreover, compared to that observed in the MMP^+^ group, the cell apoptosis rate was notably reduced in the Sp1 knockdown group, and the protective effect of Sp1 knockdown on cell viability was almost completely abolished by LRRK2 overexpression (Figure 8C, 8D). We also performed TUNEL staining (Figure 8E) and CCK-8 assays (Figure 8F) and confirmed the neuroprotective effect of shSp1, which was effectively inhibited by LRRK2 overexpression. Consistent with these findings, we observed that shSp1 significantly enhanced TUBB3 expression in MPP^**+**^-treated cells, and this effect was efficiently counteracted by LRRK2 overexpression (Figure 8G). Furthermore, we performed cell cycle analysis through propidium iodide staining and flow cytometry, the results of which further confirmed the interactive effects of shSp1 and LRRK2 on MPP**^+^-**induced cellular damages (Figure 8H, 8I). Taken together, these results provide evidence that shSp1 has a neural protective role against MPP**^+^-**induced cellular defects, which could be inhibited by LRRK2 overexpression.

### XIST knockdown or miR-199a-3p overexpression attenuates symptom severity in a PD mouse model

**Figure 9:**
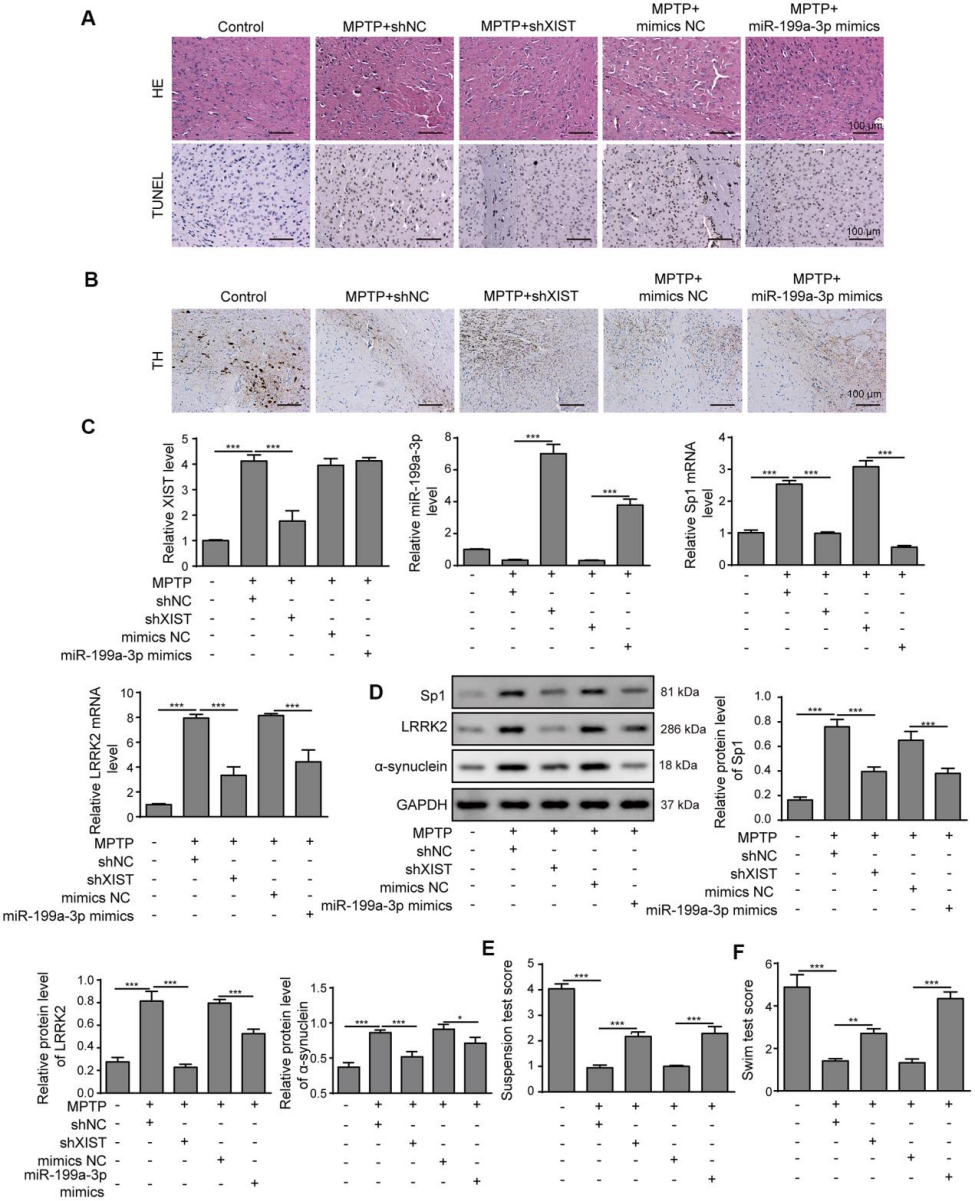
**XIST knockdown or miR-199a-3p overexpression attenuates symptom severity in PD mouse models.** (**A**) Representative microscopic images showing brain damage in the control, MPTP+shNC, MPTP+shXIST, MPTP+ mimics NC and MPTP+miR-199a-3p mimics groups. Cell apoptosis in the mouse brain tissues was detected by TUNEL staining. (**B**) Dopaminergic neuronal injury was assessed by TH immunohistochemistry. (**C**) Relative expression of XIST, miR-199a-3p, Sp1 mRNA and LRRK2 mRNA in the indicated groups was determined by qPCR. (**D**) Western blot results showed that the protein expression of Sp1, LRRK2 and α-synuclein in the PD mice was reduced by shXIST or miR-199a-3p overexpression. Animal behavioural tests, including (**E**) suspension tests and (**F**) swim tests were performed, and the scores obtained for the indicated groups are shown. The data are representative of three experiments. *p <0.05, **p <0.01 and ***p <0.001.

To assess whether our above findings are relevant in mice, we established PD animal model using MPTP. As shown in Figure 9A, mice treated with MPTP alone showed the greatest brain injuries, whereas the MPTP+shXIST and MPTP+miR-199a-3p mimics groups displayed alleviated injuries. We also assessed the dopaminergic neuronal injury by performing immunohistochemistry using a tyrosine hydrolase (TH)-specific antibody and observed that treatment with shXIST or miR-199a-3p mimics clearly enhanced TH expression that was decreased by MPTP (Figure 9B). To more conclusively show the crucial roles of shXIST and miR-199a-3p in PD animal models, we next measured XIST, miR-199a-3p, Sp1 and LRRK2 expression by qPCR. As shown in Figure 9C, miR-199a-3p expression was downregulated in our MPTP-induced animal models, whereas that of XIST, Sp1 and LRRK2 were significantly upregulated compared to that observed in the healthy controls. These effects were reversed in the MPTP+shXIST and MPTP+miR-199a-3p groups. Notably, shXIST treatment resulted in the efficient inhibition of XIST, Sp1 and LRRK2 expression but had a promoting effect on that of miR-199a-3p. In contrast, the miR-199a-3p mimics treatment increased miR-199a-3p expression and inhibited that of Sp1 and LRRK2, while XIST expression remained unchanged. Consistent with these findings, we observed a similar impact of XIST and miR-199a-3p on Sp1, LRRK2 and α-synuclein protein expression (Figure 9D). We subsequently performed behavioural analyses, including suspension and swim tests, and the PD mouse models that were treated with MPTP had significantly lower suspension and swim test scores than those observed in the healthy controls. Excitingly, the mice treated with the shXIST or miR-199a-3p mimics had much higher scores than those treated with MPTP in both tests (Figure 9E, 9F). These results indicated that XIST inhibition and miR-199a-3p overexpression play important protective roles in PD pathogenesis.

## DISCUSSION

PD is a complex neurodegenerative disease for which current treatments can only alleviate the symptoms rather than being able to prevent or reverse disease progression. The development of curative PD therapeutic approaches has been greatly hindered by our limited knowledge about the aetiology of PD. In the present study, we generated *in vivo* and *in vitro* PD models using MPTP and its toxic metabolite MPP^**+**^. Our results showed that lncRNA XIST induced Sp1 expression by sponging miR-199a-3p, and Sp1 further accelerated PD progression by targeting LRRK2. Our results elucidated the regulatory mechanisms underlying the lncRNA XIST/miR-199a-3p/Sp1/LRRK2 pathway during PD progression, and this axis represents a promising therapeutic target for PD.

MiR-199a-3p belongs to miR-199 family and was previously demonstrated to display a tumour suppressor function in various types of cancer [29–31]. However, whether and how miR-199a-3p is involved in the progression of neurodegenerative diseases, such as PD and Alzheimer’s disease, remained unclear, with no relevant research data on the topic to date. In the present study, we reported the downregulation of miR-199a-3p in both cellular and animal models of PD compared to that observed in the healthy controls. Moreover, for the first time, we demonstrated that miR-199a-3p overexpression greatly inhibits MPP^+^-induced cell apoptosis and cell cycle arrest at G1 phase, whereas miR-199a-3p inhibitor enhanced MPP^+^-induced cell apoptosis and further caused cell cycle arrest at G1 phase in the MPP^+^ cell model. MiR-199a-3p treatment effectively alleviated PD symptom in our animal models, and we showed the potential of our miR-199a-3p-targeting treatment to efficiently alleviate PD phenotypes.

In the present, for the first time, we showed that lncRNA XIST is upregulated during PD progression. Furthermore, we provided evidence that XIST modulates PD progression by targeting miR-199a-3p, and miR-199a-3p expression was upregulated by XIST knockdown. Our bioinformatics, luciferase reporter assay and RIP results further substantiated the direct binding between XIST and miR-199a-3p. Intriguingly, we observed that shXIST transfection inhibited cellular apoptosis, prevented cell cycle arrest, and promoted cell proliferation as well as TUBB3 expression in MPP^+^-treated SH-SY5Y and PC-12 cells. Notably, mice treated with shXIST displayed attenuated brain injuries and obtained much higher scores in behavioural tests than those treated with MPTP alone. However, these benefits of shXIST treatment were almost completely abrogated when the cells were simultaneously treated with miR-199a-3p inhibitor, corroborating our assertion that XIST has an important role during PD development by tightly regulating the expression and function of miR-199a-3p. Furthermore, shXIST or miR-199a-3p mimics greatly restored the TH expression that was decreased by MPTP treatment and efficiently rescued dopaminergic neuronal injury in PD animal models. Our results revealed the aberrant expression of XIST in PD models and demonstrated, for the first time, that XIST can accelerate PD progression by directly inhibiting miR-199a-3p expression. As mentioned in Introduction section, it doesn’t deny that other lncRNAs such as NEAT1, HOTAIR, SNHG1, MALAT1 and p21 and other miRNAs such as miR-128, miR-7, miR-27a/27b and miR-200a were involved in PD progression, our study mainly focused the relationship between XIST and miR-199a-3p and the networks of other lncRNAs and miRNAs will be worthy of further investigation in our future study.

Sp1 is known as an activator or a repressor in many cellular processes and plays important roles in PD-related neuropathology by modulating LRRK2 transcription and translation [17]. However, whether Sp1 has neural protective or neurotoxic roles in PD has remained controversial due to the existence of contradictory results [15, 16, 18]. For example, Ye et al. observed that Sp1 expression was upregulated in MPP^+^-treated PC-12 cells, and the oxidative stress caused by MPP^+^ was markedly reduced following the inhibition of Sp1. However, Liu and colleagues showed that Sp1 exerted protective function by attenuating LRRK2 mutant-induced PD-like symptoms. In the present study, we observed that Sp1 was upregulated in our PD cellular model and could be effectively suppressed by miR-199a-3p overexpression in both *in vitro* and *in vivo* assays. Our luciferase reporter assay results showed that miR-199a-3p directly targets Sp1. Moreover, we showed that Sp1 silencing attenuated MPP^**+**^-induced cell apoptosis and cell cycle arrest and promoted cell proliferation and TUBB3 expression in MPP^+^-treated SH-SY5Y and PC-12 cells. Strikingly, miR-199a-3p knockdown reversed these beneficial effects and further increased Sp1 and LRRK2 expression. These findings confirmed our speculation that miR-199a-3p is an upstream inhibitor of Sp1 and has a protective function against MPP^**+**^-induced neurodegenerative effects. For the first time, we demonstrated that miR-199a-3p accelerates PD progression by negatively regulating Sp1 expression. Importantly, Sp1 overexpression completely reversed the neuroprotective effects of shXIST in our PD cellular model, further showing the neurotoxic role of Sp1 in PD pathogenesis.

The results of our present study also showed that LRRK2 expression was upregulated in the PD models. Additionally, we demonstrated that LRRK2 overexpression greatly suppressed the neural protective function of shSp1. Consistent with previous reports [5, 7, 14, 17], the results of our present study suggested that Sp1 and LRRK2 are important mediators of neuronal toxicity during PD progression. The presynaptic neuronal protein α-synuclein is essentially disordered, and its aggregation is believed to be a primary cause of neuronal death in PD [40–42]. Also, previous studies indicated LRRK2 kinase regulates α-synuclein propagation in PD [43, 44]. However, the extent to which α-synuclein is implicated in PD remains obscure. In the present study, we observed that shXIST or miR-199a-3p mimics treatment greatly decreased α-synuclein expression both *in vitro* and *in vivo.*

**Figure 10:**
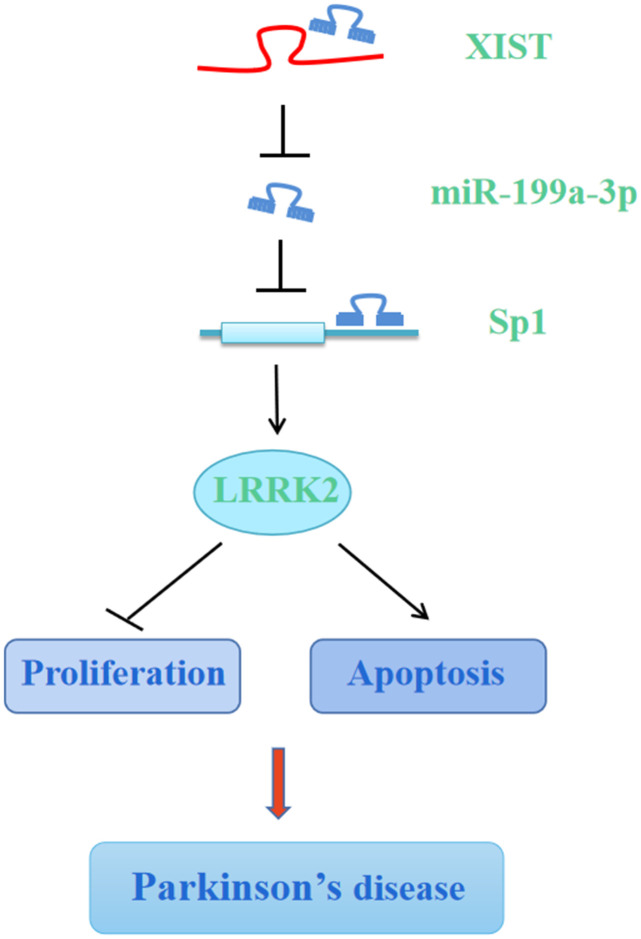
**Graphic depiction of the conclusion from the present study.** LncRNA XIST sponges miR-199a-3p to upregulate Sp1/LRRK2 axis to accelerate PD progression by inhibition of cell proliferation and promotion of cell apoptosis.

Taken together, the results of our present study showed that miR-199a-3p functions as a direct downstream molecule of XIST, and silencing XIST or miR-199a-3p overexpression attenuates MPP**^+^/**MPTP-induced cellular toxicity by downregulating Sp1 and LRRK2, suggesting that XIST and miR-199a-3p play important roles in the neurodegeneration induced by PD progression (Figure 10). In this study, we have carried out some innovations in ideas: (1) We reported for the first time the differential expression of miR-199a-3p in PD and elucidated its regulatory function during PD progression. (2) We first identified and proved the direct interaction between lncRNA XIST and miR-199a-3p by performing bioinformatics analysis, luciferase reporter assay, and RIP assay. (3) We revealed firstly the direct regulation between miR-199a-3p and Sp1 in PD. (4) Lastly, we conducted animal experiment and provided the first evidence for neuroprotective role of shXIST and miR-199a-3p overexpression in PD mouse model. By elucidating the central regulatory function of XIST and miR-199a-3p in regulating PD pathogenesis, our results offer insights into the underlying mechanisms of PD aetiology and may broaden our therapeutic perspective towards more efficient clinical treatments for PD.

## MATERIALS AND METHODS

### Cell culture and treatment

The human neuroblastoma cell line SH-SY5Y and the rat adrenal pheochromocytoma cell line PC-12 were purchased from the American Type Culture Collection (ATCC, Manassas, VA, USA) and cultured in DMEM (Gibco, CA, USA) and RPMI-1640 medium (Gibco, CA, USA), respectively, according to standard mammalian cell culture and aseptic techniques. MES23.5 dopaminergic cells were derived after the fusion of mouse neuroblastoma-glioma N18TG2 cells with rat mesencephalic neurons and were maintained in DMEM/F-12 (Gibco, CA, USA) supplemented with Sato’s components. HEK-293T cells (ATCC, Manassas, VA, USA) were cultured in DMEM for luciferase reporter assays. All media were supplemented with 10% foetal bovine serum (FBS, Gibco, CA, USA), 1% penicillin (Invitrogen, CA, USA) and streptomycin (Invitrogen, CA, USA). The SH-SY5Y and PC-12 cells were treated with 0, 0.1, 0.2, 0.4, 0.6, 0.8 or 1 mM MPP^+^ (Sigma, St. Louis, MO, USA), and PD cellular models were generated by treating the cells with 1 mM MPP^+^ for 48 h.

### Cell transfection

The miR-199a-3p mimics, miR-199a-3p inhibitor, Sp1 overexpression vector, LRRK2 overexpression vector, shSp1, shXIST and shNC were designed and synthesized by Sangon Biotech (Shanghai, China). Sp1 and LRRK2 were cloned into the vector pcDNA3.1 (+). For animal experiments, shXIST and miR-199a-3p mimics were subcloned into the vector pLKO.1. HEK-293T cells were transfected with the psPAX2 packaging plasmid, the pMD2.G envelop plasmid and the recombinant pLKO.1-shXIST or pLKO.1-miR-199a-3p plasmid using Lipofectamine 3000 (Invitrogen, CA, USA). The supernatants were harvested after 72 h, and the lentiviruses were purified and used for animal injection after the titer was determined (10^8^ PFU/mL).

### Cell proliferation determination by cell counting kit-8 (CCK-8) assay

SH-SY5Y and PC-12 cells were treated as indicated and seeded at a density of 4000 cells/well in a 96-well plate. Then, 10 μL of CCK-8 reagent (Dojindo, Kumamoto, Japan) was added to each well, and the samples were incubated for 2 h at 37° C. Then, the plate was placed at a microplate reader (Tecan Infinite 200 Pro, Switzerland), and the absorbance at 450 nm was measured, according to the manufacturer’s instructions.

### Cell cycle analysis using propidium iodide (PI) staining and flow cytometry

Cells were harvested and washed in PBS. Then, as previously described [43], 70% ethanol was added dropwise to the single cell suspensions while vortexing the cells gently, after which the cells were fixed for 1 h at 4° C. Subsequently, after being washed twice with PBS, the cells were pelleted, and 50 μL of an RNase A solution was added to a final concentration of 0.5 μg/mL. Finally, 200 μL of PI (Sigma) staining solution was added to the well-mixed cells to a final concentration of 50 μg/mL. The samples were then stored at 4° C overnight and analysed by flow cytometry.

### Cell apoptosis analysis by annexin-V/PI staining

Cell apoptosis was assessed using a FITC Annexin V/Dead Cell Apoptosis Kit from Invitrogen. Cells were harvested, washed twice in PBS, and then resuspended in 1× Annexin-binding buffer to a final density of 1×10^6^ cells/mL. Subsequently, 5 μL of FITC Annexin V and 1 μL of the 100 μg/mL PI working solution were added to each 100 μL volume of cell suspension. Finally, after incubating at room temperature for 15 min, 400 μL of 1× Annexin-binding buffer was added to each sample, and the samples were immediately analysed by flow cytometry.

### Apoptosis detection by TdT-mediated dUTP-biotin nick end labelling (TUNEL) assay

Cell apoptosis was assessed by identifying cells that underwent extensive DNA degradation using a TUNEL Kit from KeyGen BioTech (Jiangsu, China). Briefly, after the cell samples were washed 3 times with PBS, 100 μL of Proteinase K solution (90 μL PBS:10 μL Proteinase K) was added. The samples were then mixed well, incubated for 30 min at 37° C, and washed three times with PBS. Subsequently, 50 μL of the TdT enzyme solution (45 μL of Equilibration Buffer, 1.0 μL of biotin-11-dUTP, and 4.0 μL of TdT enzyme) was added to the mixtures, which were then incubated at 37° C for 60 min in darkness. After being washed with PBS for 3 times, 5 μL of a Streptavidin-Fluoresein solution and 45 μL of labelling buffer were added to the samples, which were then incubated at 37° C for 30 min in dark before being examined by fluorescence microscopy (Zeiss, Germany).

### Immunofluorescence labelling of tubulin β3 (TUBB3)

MES23.5 cells were seeded onto coverslips that were pre-coated with poly-L-lysine at room temperature. Then, the cells were fixed with 4% paraformaldehyde for 10 min and then permeabilized with 1% Triton X-100 in PBS, after which nonspecific binding was blocked with 1% bovine serum albumin (BSA). Then, the cells were incubated with a primary antibody against TUBB3 (1:50, Sigma) in 1% BSA overnight at 4° C, which followed by an incubation with an Alexa-488 conjugated secondary antibody (1:500, Invitrogen) at room temperature for 1 h. The coverslips then were washed three times with PBS, and the samples were imaged under a microscope (Zeiss, Germany).

### Luciferase reporter assay

The potential binding sites between XIST and miR-199a-3p were analysed using the DIANA web tool (http://carolina.imis.athena-innovation.gr/diana_tools/web/index.php?r=lncbasev2/index-predicted), and the binding sites between miR-199a-3p and Sp1 were predicted using TargetScan web tool (http://www.targetscan.org/vert_72/) by entering “miR-199a-3p” in the miRNA search module. The putative binding site was mutated using a QuickChange site-directed mutagenesis Kit (Stratagene, China). The XIST-WT and Sp1-WT constructs and their mutants XIST-MUT and Sp1-MUT were inserted to the luciferase reporter vector pMIR-report. Cells were seeded into a 12-well plate, grown to 80% confluence and then co-transfected with Renilla luciferase vector, pMIR-XIST-WT or pMIR-XIST-MUT; pMIR-Sp1-WT or pMIR-Sp1-MUT; and miR-199a-3p mimics, inhibitor oligos or the negative control using Lipofectamine 3000 (Invitrogen, CA, USA). After 48 h, the cells were lysed, and luciferase activities were determined using a Dual-Luciferase Reporter Assay System (Promega) according to the manufacturer’s protocols. The firefly luciferase activity values were normalized to Renilla activity in the same lysates.

### RNA immunoprecipitation (RIP) analysis

To further confirm the specific association between XIST and miR-199a-3p, an RIP assay was performed using an EZ-Magna RIP RNA-Binding Protein Immunoprecipitation Kit (Millipore, MA, USA) according to the manufacturer’s instructions. Briefly, HEK-293T cells were transfected with lentiviruses carrying miR-199a-3p mimics. Subsequently, 48 h after transfection, the cells were harvested and lysed in RIPA lysis buffer. Then, the cell lysates were incubated with magnetic beads that were conjugated with a human anti-argonaute2 (Ago2) antibody (1:50, Millipore) or the control human IgG antibody (Millipore) at 4° C for 10 h. The beads were then washed and incubated with Proteinase K buffer to degrade contaminating proteins, after which the immunoprecipitated RNA was isolated and analysed by qPCR.

### Tyrosine hydroxylase (TH) detection by immunohistochemistry

Dopaminergic neuronal cell levels were assessed by immunohistochemistry using TH specific antibody. Briefly, brain tissue samples were fixed in 4% paraformaldehyde and cut into 5 μm thick sections using a cryostat. Sections were incubated with 5% BSA for 1 h to block the non-specific bindings. Subsequently, diluted primary antibody against TH (1:1000, Abcam, UK) was applied to the samples, followed by overnight incubation at 4° C. Sections were thoroughly washed with TBST and incubated with HRP-conjugated secondary antibody for 1 h. Hematoxylin was used for nuclear counterstain and images were acquired under a light microscope.

### Animal models and grouping

The animal experimental procedures were approved by the Ethical Committee of Second Xiangya Hospital of Central South University (Changsha, Hunan, China) and were carried out in strict accordance with the institutional guidelines and ethical regulations. Forty male C57BL/6 mice (ranging from 10-12 weeks of age, mean weight of 24 g) were obtained from the SLAC Laboratory Animal Company, Ltd. (Shanghai, China). The mice were housed in individually ventilated cages with unlimited access to food and drinking water and were randomly assigned into five groups: the Control, MPTP+shNC, MPTP+shXIST, MPTP+mimics NC, and MPTP+miR-199a-3p mimics groups. The control group was treated with PBS for two weeks, and the mice in the experimental groups were intraperitoneally administered MPTP for five consecutive days (30 mg/kg/day). Mice displayed the onset of Parkinsonism after the fifth injection and were thereafter injected with lentiviral vectors carrying shXIST and miR-199a-3p mimics via tail vein for a week. Subsequently, the mice were anaesthetized with 5% ketamine, killed, and the hippocampal tissues were excised.

### Brain tissue injury assessment by haematoxylin and eosin (H&E) and TUNEL staining

The formalin-fixed, paraffin-embedded tissue sections were flamed on a burner for dewaxing and deparaffinized by 3 changes of xylene, which was followed by hydration in decreasing concentrations of alcohol (100, 90, 80, and 70%). Then, the tissue sections were stained in haematoxylin for 3 min and washed three times with distilled water before being treated with 1% acid alcohol. Subsequently, a 0.2% alkaline solution was added dropwise, after which the tissue samples were washed in distilled water and then stained in 0.5% eosin for 5 min. The sections were then thoroughly washed with distilled water, naturally dried at room temperature, and observed under a microscope (Zeiss, Germany).

The apoptotic cells in the paraffin-embedded tissue sections were labelled using a TUNEL Kit from Roche (*In Situ* Cell Death Detection kit, TMR red) according to the manufacturer’s instructions. Briefly, the TUNEL reaction mixture was prepared by adding 50 μL of Enzyme Solution to 450 μL of Label Solution. Then, the tissue sections were dewaxed and rehydrated as described above, which was followed by an incubation in a Proteinase K working solution for 20 min at 37° C. The slides were then washed with PBS, after which 50 μL of TUNEL reaction mixture was added to each sample, while 50 μL of Label solution was added for the negative control. The samples were incubated in a humidified atmosphere in the dark for 1 h at 37° C, washed three times with PBS, and then examined under a fluorescence microscope.

### Behavioural experiments

### Tail suspension test

Mice were transferred to a rectangular tail suspension box (60×40×15 cm) by wrapping their tails to a horizontal wire 30 cm from the base. The number of attempts made per minute to clasp the wire with hind limbs was measured on a score from 1 to 3, where 1= no clasping, 2= clasping using one hind limb, and 3= clasping using both hind limps.

### Swim test

Mice were gently picked by the tail and transferred into a cylindrical tank (30×20×20 cm) filled with water (25±1° C) to the level of 15 cm. Timer was set at 6 min and started to record the mouse mobility. The behavioural parameter was scored as follows: 3 points for mice that could non-stop swim during the test, 2.5 points for mice that mostly swam and occasionally floated, 2 points for mice that mostly floated during the test (>3 min), 1.5 points for mice that occasionally swam, and 1 point for mice that occasionally moved their hind limbs.

### RNA extraction and qPCR

Hippocampal tissues or cell lines were homogenized, and total RNA was extracted using TRIzol reagent (Invitrogen) according to the manufacturer’s instructions. The purity and concentration of the total RNA samples were determined by UV spectrometry. RNA was reverse transcribed using random hexamer primers with a TAKARA Reverse Transcriptase Kit (Takara, Dalian, China), and SYBR Green qPCR Master Mix (Takara, Dalian, China) was used to perform qPCR. GAPDH and U6 small nuclear RNA (U6 snRNA) were used as endogenous expression references, and relative expression was calculated using the 2^−ΔΔCt^ method. The primers used for XIST, miR-199a-3p, Sp1, LRRK2 and α-synuclein were synthesized by Sangon Biotech (Shanghai, China).

### Western blot analysis

Western blot analysis was performed as previously described [8]. Cells were harvested and lysed in ice-cold RIPA lysis buffer, which contained protease inhibitor cocktail (Sigma). Protein concentration was measured using Bradford Protein Assay Kit (Beyotime, China). Subsequently, 30 μg of total protein samples from the cleared lysates were separated on a 10% sodium dodecyl sulfate polyacrylamide gel (SDS-PAGE) and then electrotransferred to nitrocellulose membranes. The membranes were blocked with 1% BSA for an hour and then further incubated with primary antibodies against Sp1 (1:1000, Abcam), LRRK2 (1:1000, Abcam), α-synuclein (1:2000, Abcam) or GAPDH (1:1000, Cell Signaling Technology) at 4° C overnight. The following day, the blots were washed three times in TBST buffer and then incubated with horseradish peroxidase-conjugated secondary antibodies for 1 h at room temperature. The blots were scanned with an Odyssey infrared scanner (Li-Cor Biosciences Inc.), and protein levels were quantified using ImageJ.

### Statistical analysis

All experiments were conducted independently more than three times. SPSS 20.0 was used to analyse data, and the data were presented as the means ± standard deviation (SD). Comparisons between two groups were performed using Student’s t-test. Comparisons among three or more groups were conducted using one-way analysis of variance (ANOVA) followed by Tukey’s post hoc test. A value of *P*<0.05 was considered to indicate a significant difference for all analyses.

## Supplementary Material

Supplementary Figures
