# Supplementary material for: LncRNA XIST sponges miR-199a-3p to modulate the Sp1/LRRK2 signal pathway to accelerate Parkinson’s disease progression
Source: Aging (Albany NY). 2021 Jan 20;13(3):4115–37. doi: 10.18632/aging.202378 (PMC7906184; doi:10.18632/aging.202378)
Supplement: Supplementary Figures [file aging-13-202378-s001.pdf]

SUPPLEMENTARY FIGURES

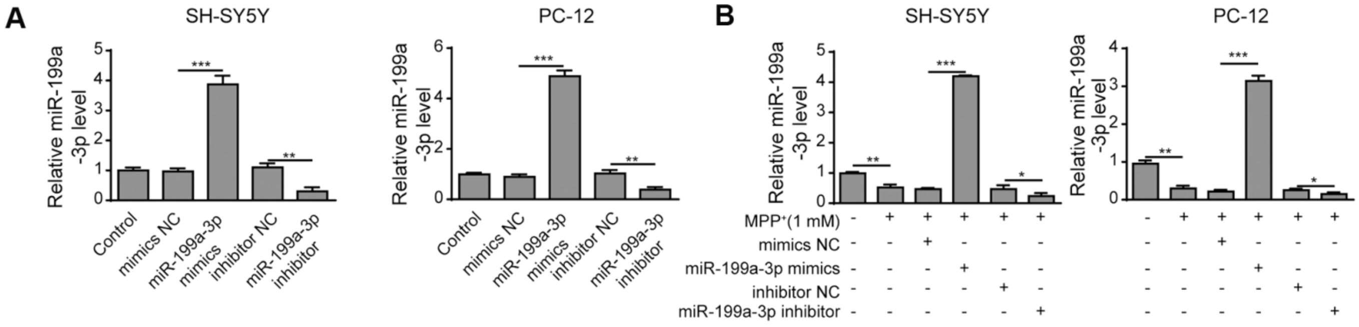

Supplementary Figure 1. (**A**) SH-SY5Y and PC-12 cells were transfected with miR-199a-3p mimics or the miR-199a-3p inhibitor. qPCR was conducted to determine the transfection efficiency. (**B**) SH-SY5Y and PC-12 cells were treated with 1 mM MPP^+ and transfected with the miR-199a-3p mimics, miR-199a-3p inhibitor, mimics NC or inhibitor NC. The relative expression of miR-199a-3p was determined by qPCR analysis.

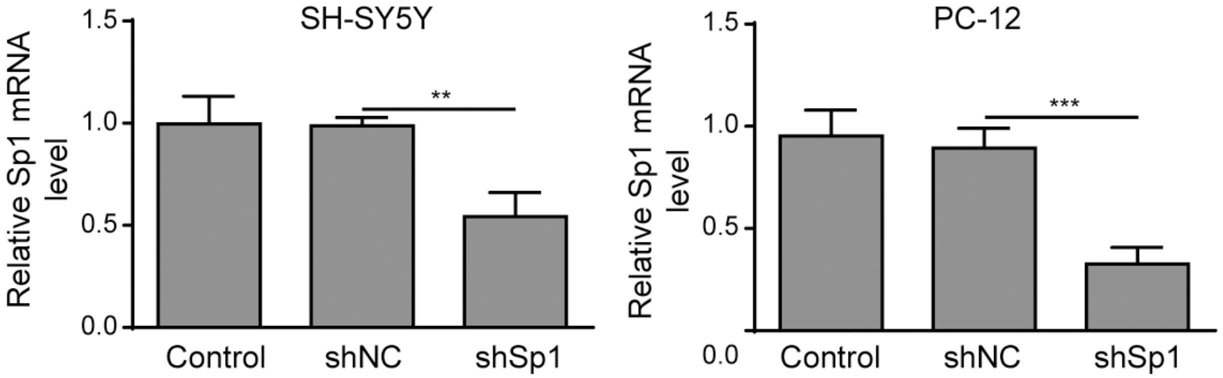

Supplementary Figure 2. SH-SY5Y and PC-12 cells were transfected with shNC or shSp1, and transfection efficiency was determined by qPCR.

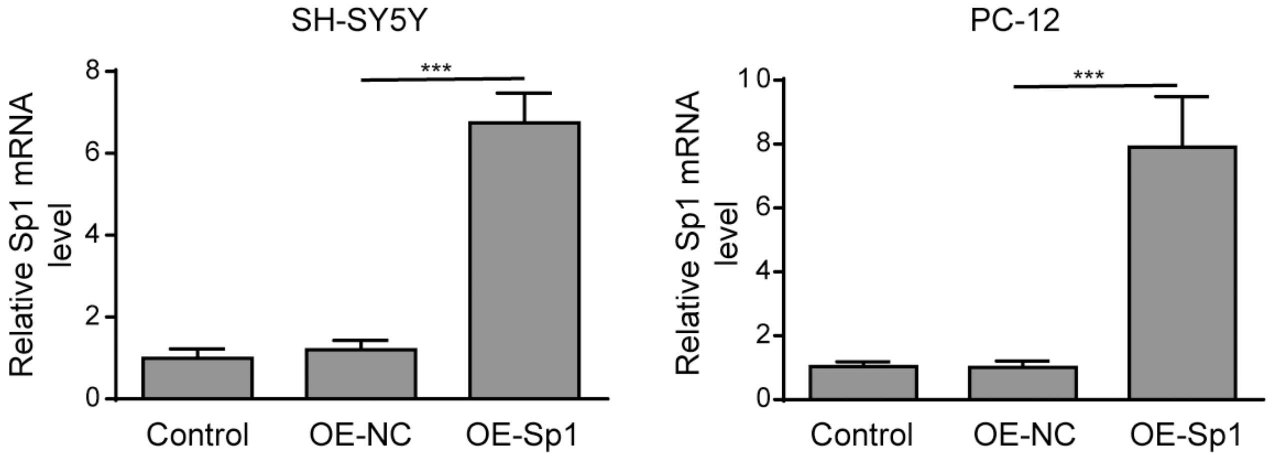

Supplementary Figure 3. SH-SY5Y and PC-12 cells were transfected with OE-NC or OE-Sp1, and transfection efficiency was determined by qPCR. OE: Overexpression.

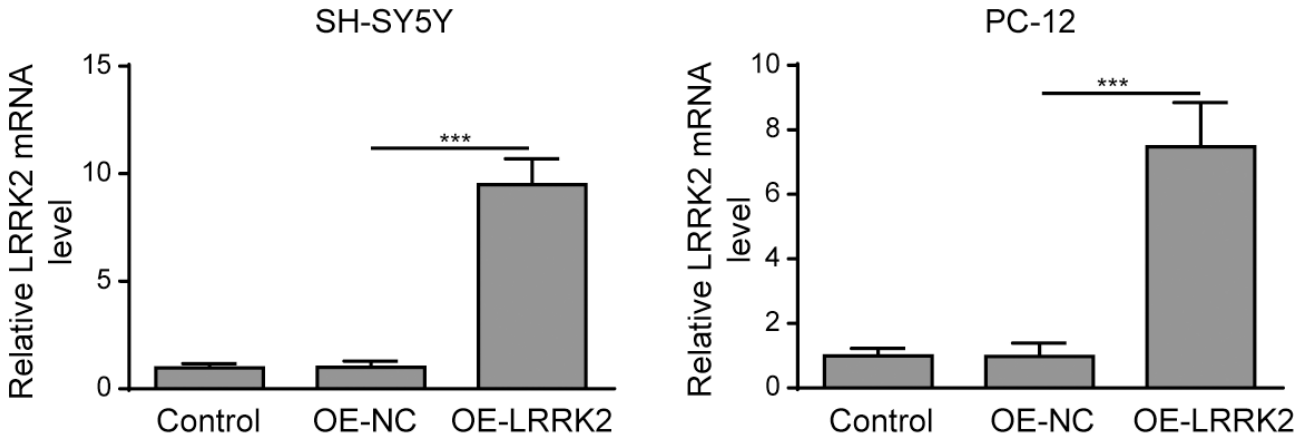

Supplementary Figure 4. SH-SY5Y and PC-12 cells were transfected with OE-NC or OE-LRRK2, and the transfection efficiency was measured by qPCR. OE: Overexpression.
